# Novel Hypothesis on Phase Angle as a Candidate Marker Associated with Physiological Reserve in Geriatric Patients in Long-Term Care

**DOI:** 10.3390/geriatrics11050113

**Published:** 2026-08-25

**Authors:** Alejandro Padilla Isassi, Rosalba Maya Hernández, María Elisa Otero Cerdeira, Ingrid Alejandra Abarca Salinas

**Affiliations:** 1 Geriatrics Department and Research Area, Hospital Español, Av. Ejército Nacional Mexicano 613, Granada, Miguel Hidalgo, Mexico City 11520, Mexico; 2 Department of Clinical Nutrition, Hospital Español, Av. Ejército Nacional Mexicano 613, Mexico City 11520, Mexico; nut.rosalbam@gmail.com; 3 Neurology Department, Hospital Español, Mexico City 11520, Mexico; sommahespanol@gmail.com; 4 Research Area, Hospital Español, Av. Ejército Nacional Mexicano 613, Mexico City 11520, Mexico; ingrid.abarcasa@gmail.com

**Keywords:** phase angle, physiological reserve, frailty, sarcopenia, functional status

## Abstract

**Highlights:**

- Phase angle as a candidate biomarker of physiological reserve in long-term-care residents.
- Phase angle is associated with common diseases in long-term care older adults.
- Multifrequency bioelectrical impedance aids assessment of older adults.
- Bioimpedance assessment may be the future of geriatric evaluation.

**Abstract:**

**Background**: Physiological reserve reflects the capacity of the organism to maintain homeostasis under biological stress. We suggest the hypothesis that phase angle derived from bioelectrical impedance analysis could be a potential objective marker of Physiological reserve. **Methods**: An analytical cross-sectional study was conducted in 45 older adults residing in long-term care units at Hospital Español de México (2025). Frailty was assessed with the FRAIL index, sarcopenia using EWGSOP2 criteria, functionality with the Barthel Index, and quality of life with WHOQOL-OLD. Body composition and phase angle were measured by multifrequency bioelectrical impedance (InBody BWA 2.0^®^). Associations were analyzed using ROC curves and regression models. **Results**: Phase angle (PA) was closely associated with key geriatric domains. Frail participants showed significantly lower PA values (3.1 ± 0.7 vs. 3.8 ± 0.9; *p* = 0.009), while sarcopenic patients presented lower PA (3.1 ± 0.7 vs. 4.1 ± 0.9; *p* < 0.001). PA demonstrated a strong positive correlation with functional status (*r* = 0.668; *p* < 0.001). A moderate positive correlation was observed with quality of life (*r* = 0.551; *p* = 0.001). In an exploratory multivariable analysis, PA was independently associated with age (B = −0.063; *p* < 0.001), functionality (B = 0.013; *p* < 0.001), and frailty (B = −0.137; *p* = 0.044). **Conclusions**: The phase angle is associated with key geriatric conditions that are related to reduced physiological reserves in older adults in long-term care, addressing the hypothesis that phase angle reflects physiological reserve at the cellular level by providing preliminary cross-sectional evidence.

## 1. Introduction

Physiological reserve is one of the most extensively examined constructs across medical specialties involved in the care of older adults [1,2,3]. It represents a multidimensional clinical construct that reflects an individual’s degree of biological vulnerability and, consequently, informs prognostic estimation in the context of acute illness or chronic disease. In essence, physiological reserve denotes the capacity to maintain homeostasis when exposed to biological stressors [4].

Traditionally, the assessment of this reserve has been closely linked to the evaluation of vulnerability and has been conducted through approaches based on physical spectra and cumulative deficit models, such as those proposed by Fried [5] and Rockwood [6]. These approaches enable an indirect evaluation of the phenomenon and have demonstrated utility over time. Nevertheless, in a context of continuous technological advancement, there is a growing need for more objective and quantifiable measurements [7,8], which facilitate not only identification of the problem but also its clear and understandable communication to the patient.

Within this framework, bioimpedance has emerged as a technique widely employed in multiple areas of research [9,10,11,12]. Various studies have demonstrated its capacity to discriminate between clinically relevant pathologies and parameters associated with poor prognosis, although these cut-off points have been established in a non-standardized manner [13]. To date, there is no solid consensus that uniformly defines the clinical significance of these parameters.

This lack of consensus largely stems from the variability observed among the populations studied [14], whose reference values differ from one another. However, it is pertinent to question why, despite these population differences, such parameters continue to demonstrate discriminative capacity for chronic degenerative diseases. This suggests that we are not dealing solely with absolute values but rather with parameters that dynamically adjust to an underlying pathophysiological process. Consequently, it is reasonable to propose that these indicators do not merely reflect a static state but instead adapt to the progression of the biological process [11,15].

One promising indicator is the phase angle, which has been one of the most extensively studied parameters within bioimpedance. It has demonstrated high diagnostic sensitivity across multiple clinical scenarios: patients with complex comorbidities, renal, hepatic and heart failure, cancer, cardiac rehabilitation, sarcopenia, and functional decline, among others [13,16,17,18,19,20,21,22]. However, each study proposes different cut-off points, which does not represent a weakness of the parameter but rather is the logical consequence of its application in entities characterized by different degrees of inflammation, cell damage, age and stage of the aging process.

The key to understanding the phase angle lies in its bioelectrical basis. It is modeled from resistance, which reflects opposition to the passage of current through body fluids, and reactance, which derives from the “capacitor” effect [23]. An electrical capacitor is a passive component that stores energy between two conductive plates separated by an insulating material. Similarly, the cell membrane behaves as a biological capacitor.

Therefore, the phase angle reflects the integrity and functionality of the cell membrane. A lower “capacitor effect” corresponds to the reduced structural and functional integrity of the membrane, implying a diminished cellular capacity to adapt to environmental changes [24]. In physiological terms, reserve depends on how many membranes maintain this function adequately.

Aging introduces an essential temporal component. Just as an electrical capacitor loses efficiency over time, the aging cell membrane exhibits structural and functional alterations associated with cellular senescence [25]. A senescent membrane has a reduced capacity to regulate the exchange of fluids, proteins, and oxidative products, thereby decreasing cellular adaptability.

The literature consistently shows that phase angle correlates with age [15,26] and discriminates among multiple pathologies, reflecting the cumulative impact of comorbidities on cell membrane integrity and biological response capacity. In this sense, phase angle may be understood as a quantifiable expression of physiological reserve at the cellular level.

In our research, we deliberately address the most heterogeneous population: older adults with high comorbidity, in whom isolating the effect of a single variable is complex. Precisely in this context—where variability is the norm—we aim to assess whether the parameter retains its utility. Furthermore, this investigation suggests the potential clinical relevance and practical applicability of this parameter, indicating that it may remain a useful tool even under conditions of substantial biological and clinical heterogeneity.

In this cohort, we will evaluate three key entities associated with low physiological reserve and poor prognosis in geriatrics: frailty [27], sarcopenia [28], and functional dependence [29,30,31]. In addition, we will incorporate quality of life as a central outcome, particularly relevant in long-term care, considering that low physiological reserve is associated with multiple limitations that directly impact this dimension [32].

In this way, we will be able to observe how phase angle behaves in a real and heterogeneous geriatric population and assess its association to physiological reserve.

Rather than providing a definitive answer, this study seeks to begin addressing the hypothesis that phase angle reflects physiological reserve at the cellular level by providing preliminary cross-sectional evidence and laying the groundwork for future longitudinal and mechanistic research.

## 2. Materials and Methods

### 2.1. Study Design

An analytical cross-sectional study was conducted in older adults residing in long-term care facilities at the Hospital Español de México during 2025. Prior to data collection, written informed consent was verified by the participant or from their caregiver/legal guardian for the investigation and as part of the routine clinical evaluation.

Information was obtained through direct interviews with the patient and their primary caregiver during the semiannual clinical assessment established by the institutional algorithm for chronic diseases, performed by specialist physicians and including a comprehensive geriatric assessment.

Data on chronic diseases were extracted from the institutional medical records. Functional performance tests were administered on the same day, with caregiver corroboration, allowing data collection to be completed in a single visit and ensuring a comprehensive evaluation.

Clinical trial number: not applicable.

### 2.2. Population

Convenience sampling was performed, including all geriatric patients in long-term care who met the selection criteria during the study period. Of a total of 62 institutionalized older adults, 45 patients were evaluated. Patients with acute pathology, those without consent for the use of clinical information, and those meeting criteria for terminal status according to the Guía de Manejo Integral de Cuidados Paliativos en México were excluded.

### 2.3. Dependent Variables

Frailty, sarcopenia, and functional impairment were selected as primary outcomes because they are widely recognized clinical manifestations of diminished physiological reserve in older adults. Although physiological reserve cannot be directly measured through a single instrument, these domains are consistently used in geriatric research as complementary indicators of reduced biological resilience, vulnerability to stressors, and loss of homeostatic capacity. Additionally, quality of life was included as a dependent variable, as it constitutes a central objective in the care of institutionalized older adults. Unlike the former variables, it does not represent a marker of physiological reserve.

Frailty was assessed using the FRAIL index, which includes five dichotomous components: fatigue, resistance, ambulation, comorbidity, and unintentional weight loss. The presence of three or more criteria was considered indicative of frailty. This instrument was selected because it integrates the physical dimension and the accumulation of deficits, approaches that have demonstrated greater applicability in institutionalized geriatric populations [33,34].

Sarcopenia was diagnosed according to the algorithm of the European Working Group on Sarcopenia in Older People 2 (EWGSOP2). Muscle strength was measured using a Jamar^®^ hydraulic dynamometer, considering <27 kg in men and <16 kg in women as reduced strength. Skeletal muscle mass was estimated by bioelectrical impedance analysis, with low muscle mass defined as a skeletal muscle mass index (SMI) < 7.0 kg/m^2^ in men and <6.0 kg/m^2^ in women. Confirmed sarcopenia required both reduced strength and low muscle mass; the 29 participants classified as sarcopenic met both criteria.

Functional status was assessed using the Barthel Index (BI), which evaluates basic activities of daily living (feeding, hygiene, continence, mobility, and transfers). Scores range from 0 to 100, with higher values indicating greater independence.

Quality of life (QoL) was measured using the World Health Organization Quality of Life—Old Assessment (WHOQOL-OLD) questionnaire, which explores six domains: sensory abilities, autonomy, past/present/future activities, social participation, intimacy, and attitudes toward death. Each domain is scored from 4 to 20; higher scores reflect better perceived quality of life.

### 2.4. Study Covariates

During the assessment visit, the following covariates were recorded: age, sex, years in long-term care, diaper use, and multimorbidity. Multimorbidity was defined as the presence of more than five chronic diseases, identified on the same day through review of the clinical record and available medical reports. Although multimorbidity constitutes one of the components of the FRAIL index, it was included as an independent covariate due to its clinical relevance in this population [35]. In institutionalized older adults, the coexistence of multiple diseases is common; however, the presence of more than five pathologies, as established in the FRAIL index, has demonstrated greater discriminative capacity to identify subgroups at higher clinical and functional risk [36], particularly in long-term care settings.

Regarding anthropometric measurements, height was measured using an electronic stadiometer and recorded in centimeters, then corroborated with the values obtained through bioimpedance. Body weight was determined using a conventional scale in patients able to stand; in those unable to maintain an upright position, a standardized bed scale was used, subtracting the weight of the bed beforehand. Values were recorded in kilograms.

Body circumferences were measured using measuring tape and expressed in centimeters. Body mass index (BMI) was calculated using the formula: weight in kilograms divided by height in meters squared (kg/m^2^).

### 2.5. Electrical Bioimpedance

Body composition was assessed using segmental multifrequency bioelectrical impedance analysis with the InBody BWA 2.0^®^ device (InBody Co., Ltd., Seoul, Republic of Korea), under standardized conditions. Measurements were performed in the supine position after a 10 min rest period, using four clamp-type electrodes.

From a clinical perspective focused on older adults, parameters considered relevant for this population were selected for analysis. The body composition variables included were total body water (TBW), intracellular water (ICW), extracellular water (ECW), proteins, minerals, body fat mass (BFM), soft lean mass (SLM), fat free mass (FFM), skeletal muscle mass (SMM), percent body fat (PBF), body cell mass (BCM), bone mineral content (BMC), skeletal muscle index (SMI), and whole body phase angle at 50 kHz (PhA).

### 2.6. Statistical Analysis

Statistical analysis included both descriptive and inferential statistics. Variables were examined in relation to four entities defined a priori. Frailty and sarcopenia were analyzed as dichotomous variables, according to the FRAIL index and the EWGSOP2 algorithm, respectively. Functional status and quality of life were analyzed as quantitative variables.

Receiver operating characteristic (ROC) curves were constructed to evaluate bioimpedance variables in the identification of frailty and sarcopenia, supported by binary logistic regression models. Final model selection was performed using stepwise methods based on the Akaike Information Criterion (AIC) and complemented by clinical judgment. To assess the relationship between bioimpedance parameters with functional status and quality of life, linear regression models were applied. To analyze the context of phase angle, a multivariable linear regression model was constructed. Covariates were selected a priori on clinical and pathophysiological grounds rather than by automated stepwise procedures, and the model was deliberately restricted to three clinically prioritized predictors to limit overfitting given the sample size. Age was included because it is consistently associated with phase angle in the literature and is central to our hypothesis, whereas functional status (Barthel) and frailty (FRAIL) were included as clinically established expressions of diminished physiological reserve. Collinearity among covariates was assessed with Spearman and Kendall rank correlations: age was essentially uncorrelated with the other covariates (ρ ≤ 0.10), while frailty and functional status showed a moderate inverse correlation (Spearman ρ = −0.54, *p* = 0.001), consistent with their shared physical performance component. The two constructs are not redundant, however, as the FRAIL scale additionally captures comorbidity, exhaustion and weight loss that the Barthel index does not, and both retained independent, statistically significant associations with phase angle in the adjusted model. No multicollinearity of concern was therefore detected.

All confidence intervals were set at 95%, and statistical significance was defined as *p* < 0.05. The analysis was conducted using IBM SPSS Statistics, version 29.0 (IBM Corp., Armonk, NY, USA).

### 2.7. Ethical Considerations

The present study was conducted in accordance with the ethical principles established in the Belmont Report (1979), which govern research involving human subjects—respect for persons, beneficence, and justice—as well as in compliance with the Declaration of Helsinki. It was approved by the Research and Ethics Committee of the Hospital Español de México.

All institutionalized participants provide informed consent for periodic evaluations aimed at monitoring their clinical course. Additionally, patients, their family members, and primary caregivers were informed about the study. No procedures were performed outside current regulatory guidelines, as all evaluations are part of the institution’s routine clinical follow-up.

### 2.8. General Characteristics of the Population

A total of 45 patients were analyzed out of the 62 evaluated in the long-term care units. A predominance of women was observed (73.3%), with a mean age of 87.5 years (SD ± 7.9). More than half used diapers (62.2%), and 80% had a paid caregiver. The mean length of stay was 7.3 years (±5.9).

Patients with multimorbidity represented 13.3% of the sample. The mean BMI was 27.4 (±5.5), and the right and left thigh circumferences were 44.5 cm (±8.3) and 45.4 cm (±4.7), respectively. Regarding bioimpedance parameters, the sample presented a mean TBW of 28.37 L (±6.19), distributed between ICW 16.67 L (±3.71) and ECW 11.70 L (±2.53). Nonfat body components showed mean values of protein 7.21 kg (±1.61) and minerals 3.03 kg (±0.55), while SLM reached 36.04 kg (±7.89). SMM was 19.75 kg (±4.83) and BCM 23.88 kg (±5.32). BFM was 29.72 kg (±11.72), and PBF reached a high mean of 42.10% (±9.41). SMI showed a value of 5.34 (±1.48), and PhA had a mean of 3.48° (±0.87) (Table 1).

### 2.9. Clinical Results and Bioimpedance in Frailty

Of the 45 participants included, 23 (51.1%) were classified as frail. A higher proportion of men was observed in the frailty group (39.1% vs. 13.6%); however, this was not statistically significant (*p* = 0.054). Mean age did not differ significantly between groups. Diaper use was more frequent in the frailty group (78.3% vs. 45.5%; *p* = 0.024). Likewise, the presence of a paid caregiver was significantly higher among participants with frailty (95.7% vs. 63.6%; *p* = 0.009).

Anthropometric measurements showed no significant differences between groups. Mean height was comparable. Similarly, body weight and BMI did not differ significantly. No differences were identified in circumferences.

**Figure 1:**
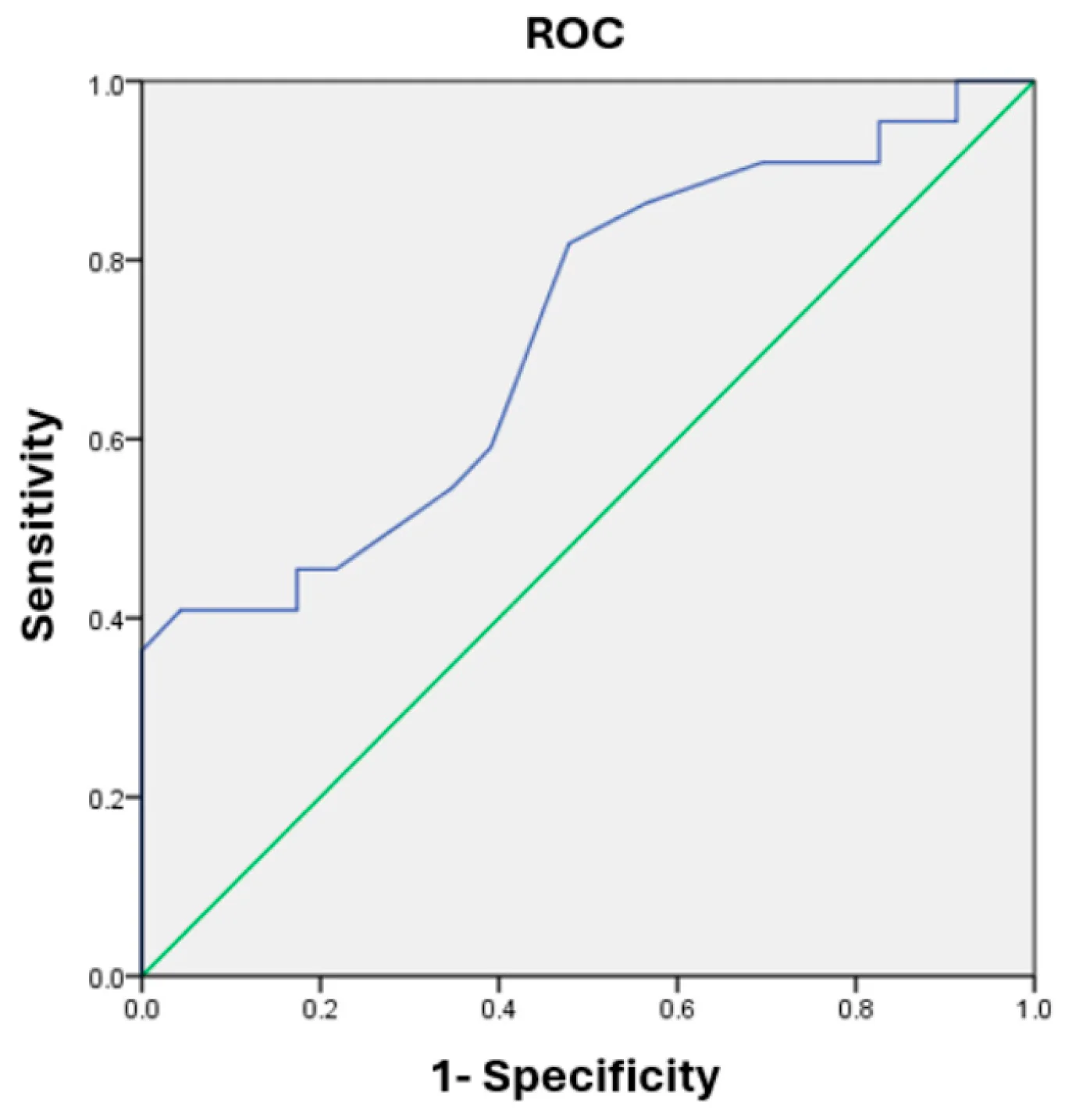
ROC curve analysis of phase angle for frailty diagnosis.

Bioimpedance parameters showed no differences in body water compartments or in components related to BFM, FFM, SMM, BCM, SMI, or BMC (Table 1). In contrast, PhA was significantly lower in the frailty group (3.1 ± 0.7 vs. 3.8 ± 0.9; *p* = 0.009). ROC analysis demonstrated an area under the curve (AUC) of 0.718. The optimal cut-off point was identified at 3.25, with a sensitivity of 81.8% and a specificity of 52.2% for frailty (Figure 1).

### 2.10. Sarcopenia

Twenty-nine participants (64.4%) met criteria for sarcopenia according to EWGSOP2. Sex distribution was similar between groups. Participants older than 80 years were more affected (96.6% vs. 62.5%; *p* = 0.005). The presence of a paid caregiver was higher among participants with sarcopenia (89.7% vs. 62.5%; *p* = 0.039).

Participants with sarcopenia showed significantly lower values in multiple anthropometric parameters. Mean height was significantly lower (154.4 ± 8.8 vs. 161.9 ± 10.4 cm; *p* = 0.013). Body weight and BMI were also lower (62.1 ± 15.9 kg vs. 79.7 ± 14.6 kg; *p* = 0.001 and 25.9 ± 5.6 vs. 30.3 ± 4.4 kg/m^2^; *p* = 0.009, respectively). Abdominal and hip circumferences were smaller (94.0 ± 19.0 vs. 107.4 ± 20.4 cm; *p* = 0.033 and 91.6 ± 7.5 vs. 99.5 ± 4.2 cm; *p* < 0.001, respectively). Likewise, extremity circumferences—including calf, thigh, and arm—were significantly lower in participants with sarcopenia (Table 1).

Regarding bioimpedance parameters, significant differences were observed in most body composition components. Participants with sarcopenia had significantly lower TBW, ICW, and ECW (*p* ≤ 0.001). Protein and mineral related components were also lower (*p* < 0.001 and *p* = 0.019, respectively).

**Figure 2:**
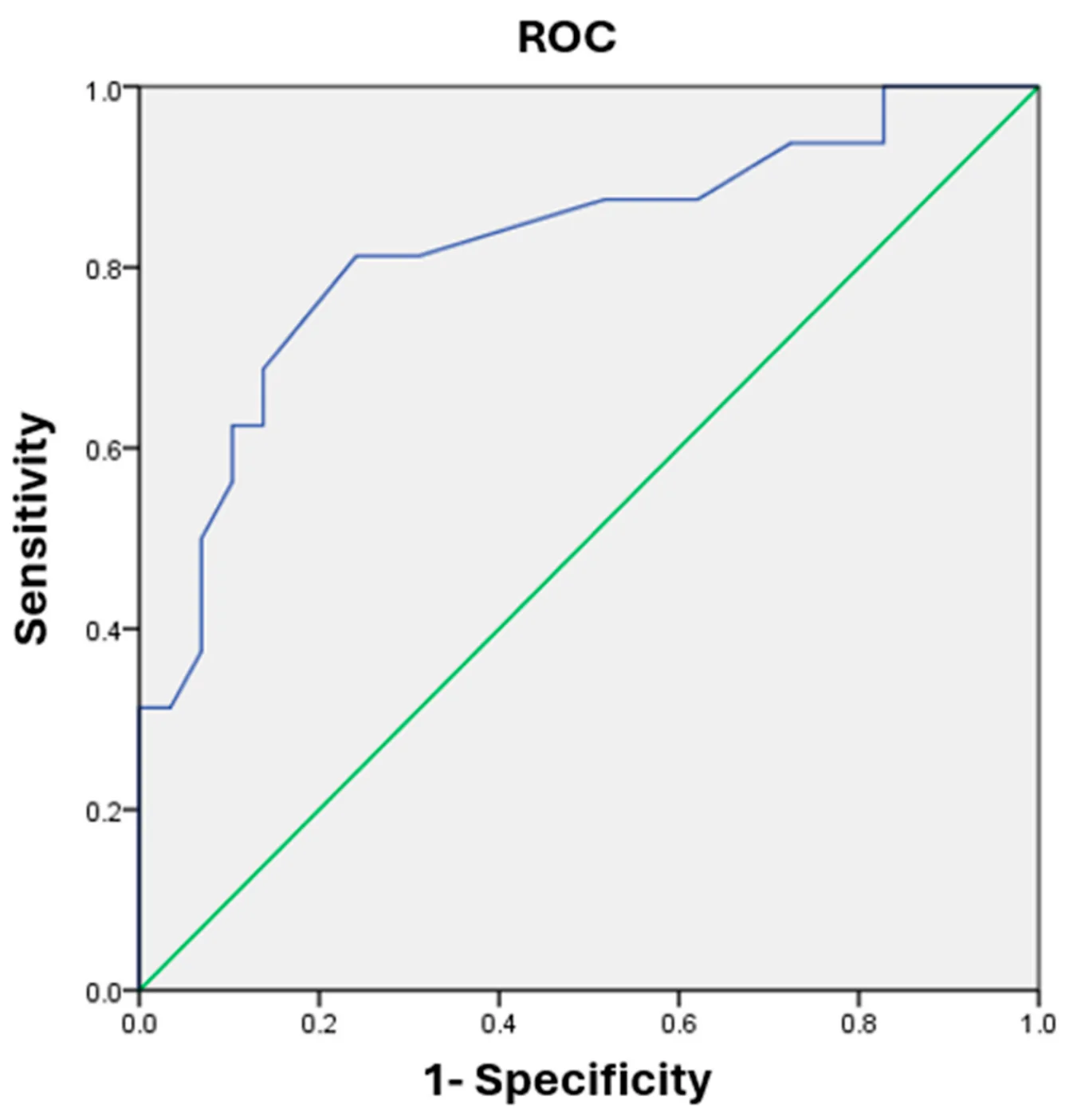
ROC curve analysis of phase angle for sarcopenia diagnosis.

In terms of body mass components, participants with sarcopenia showed lower values of SLM, FFM, and SMM (all comparisons *p* < 0.001). Similarly, with BCM, BMC and SMI. Finally, PhA was significantly lower in participants with sarcopenia (3.1 ± 0.7 vs. 4.1 ± 0.9; *p* < 0.001). An exploratory ROC analysis showed an AUC of 0.824, with a cut-off point of 3.50, sensitivity of 81.3%, and specificity of 75.9% for detecting sarcopenia (Figure 2); this threshold was derived internally and should be regarded as exploratory pending external validation.

**Figure 3:**
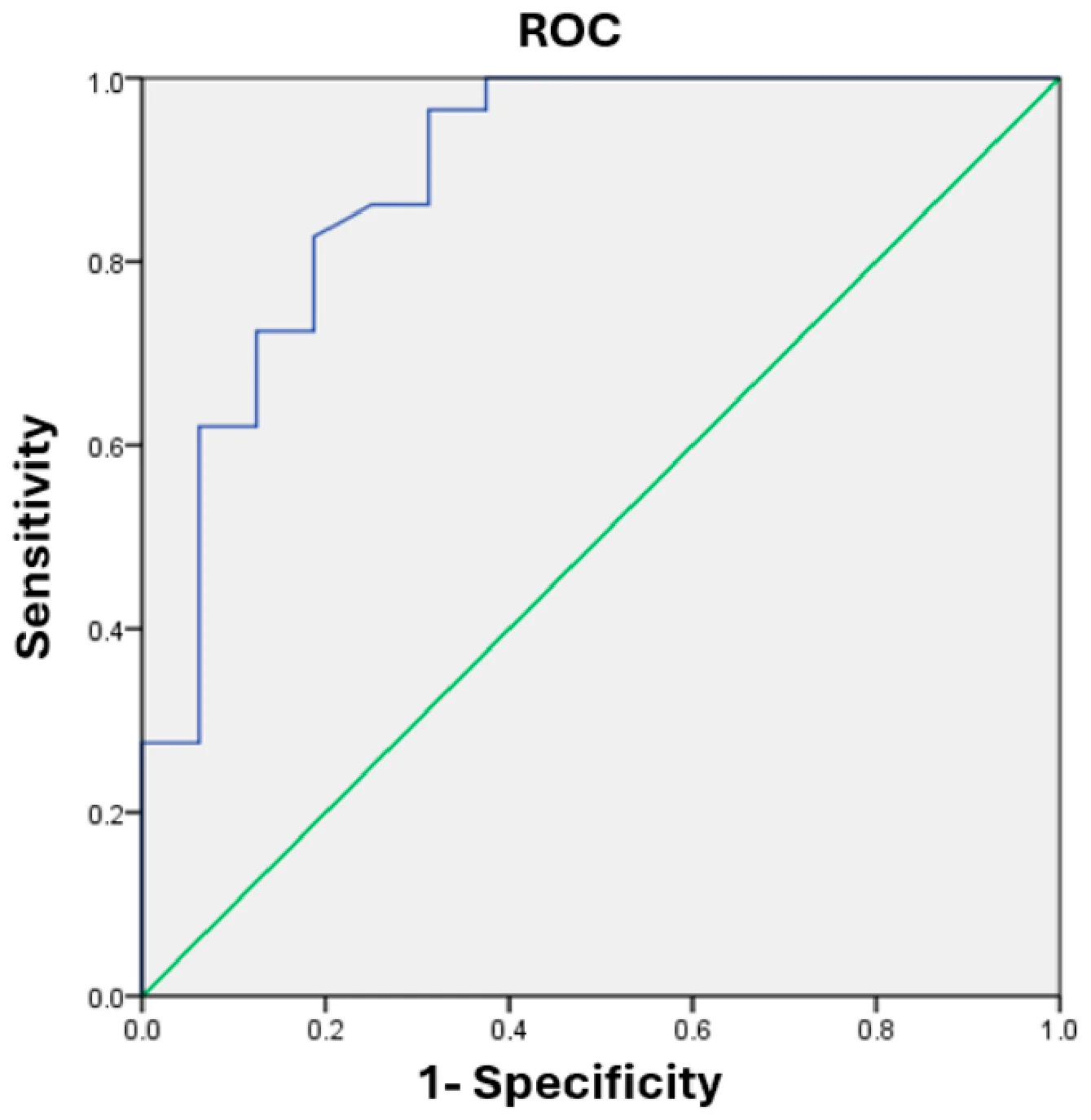
ROC curve analysis of total body cell mass and phase angle for sarcopenia detection.

BCM and PhA were adjusted using binary logistic regression, yielding an AUC of 0.893 (Figure 3) with a sensitivity of 96% and specificity of 68% for a probability ≥ 0.63, using the formula detailed in Formula 1. PhA and BCM showed only weak intercorrelation (Spearman ρ = 0.40; VIF ≈ 1.2), indicating negligible collinearity and non-redundant contributions. Nevertheless, both predictors and the BIA estimated muscle mass criterion of the EWGSOP2 label derive from the same bioelectrical signal; the combined model AUC (0.893) should therefore be regarded as an apparent, internally optimistic estimate reflecting shared measurement source, only partially attenuated by the impedance-independent handgrip component. Given the sample size (16 non-sarcopenic cases) and the absence of internal validation, this figure requires confirmation in larger cohorts.

(1) $$p=\frac{1}{1+e^{-(15.539-1.929\;\left(PhA\right)-0.331\;\left(BCM\right))}}$$

Formula (1) Linear regression equation for the prediction of sarcopenia using phase angle and body cell mass. Note: The optimal cut-off point of the predictive model was ≥0.63 probability, achieving a sensitivity of 96% and a specificity of 68%.

### 2.11. Functionality

The BI, as a measure of functionality, showed no significant differences according to sex and no correlation with age. Participants using diapers had lower BI scores (34.3 ± 18.2 vs. 80.4 ± 29.0; *p* < 0.001). Similarly, the presence of a paid caregiver was associated with lower functionality (40.0 ± 24.5 vs. 96.3 ± 6.9; *p* < 0.001), findings consistent with the clinical context of the patient.

No significant associations were observed between BI and anthropometric variables, including height, weight, and BMI. Likewise, abdominal, hip, calf, and arm circumferences did not show significant correlation with functionality. In contrast, a moderate and significant positive correlation was identified with thigh circumferences, both for right (*r* = 0.478; *p* = 0.004) and left (*r* = 0.449; *p* = 0.008) thighs.

Most bioimpedance parameters did not show significant correlations. However, a moderate and significant positive correlation was identified with SMI (*r* = 0.362; *p* = 0.036), and notably, PhA showed a strong positive correlation (*r* = 0.668; *p* < 0.001). Linear regression analysis demonstrated that for each one-unit increase in phase angle, the BI increased by an average of 24.5 points (Table 2).

### 2.12. Quality of Life

Quality of life (QoL) did not show significant differences according to sex (73.0 ± 12.4 vs. 68.3 ± 15.0; *p* = 0.427) but did differ categorically by age: participants older than 80 years had lower WHOQOL-OLD scores (68.3 ± 15.3 vs. 75.8 ± 2.6; *p* = 0.022).

Participants using diapers had lower QoL (62.8 ± 12.7 vs. 78.4 ± 11.6; *p* = 0.001). The presence of a paid caregiver was associated with lower scores (64.8 ± 13.2 vs. 83.9 ± 5.8; *p* < 0.001), as contextually expected given patient limitations.

No significant correlations were observed for most bioimpedance parameters. Only PhA showed a moderate and significant positive correlation with WHOQOL-OLD scores (*r* = 0.551; *p* = 0.001). Linear regression analysis demonstrated that for each one unit increase in PhA, QoL increased by 8.8 points (Table 2).

### 2.13. Age and Phase Angle

In the final exploratory multivariable linear regression model, PhA was the dependent variable, and age, functionality, and frailty were included as independent variables in the same model. Age showed a significant inverse association (B = −0.063; β = −0.620; *p* < 0.001; 95% CI: −0.077 to −0.048). Functionality was positively and independently associated (B = 0.013; β = 0.470; *p* < 0.001; 95% CI: 0.008 to 0.017). Frailty showed a significant negative association (B = −0.137; β = −0.176; *p* = 0.044; 95% CI: −0.270 to −0.004). In the adjusted model, age, functionality, and frailty remained independent and statistically significant predictors of PhA (Table 3).

## 3. Discussion

Bioelectrical impedance analysis has been described in multiple studies as a sensitive and clinically meaningful tool capable of discriminating between patients with different pathologies or geriatric syndromes [9,37,38]. This supports its inclusion as a component of comprehensive geriatric assessment. Nevertheless, universally accepted reference parameters have not yet been established. This heterogeneity of cut-off values may partly reflect genuine differences in aging trajectories and comorbidity burden [8,19], but it also represents a real barrier to clinical application that standardized, age and comorbidity adjusted references will need to resolve.

The present study focuses on older adults receiving long-term care, a population characterized by marked clinical complexity and heterogeneity. Despite this variability, phase angle demonstrated a pattern consistent with previous reports, aligning with the specific characteristics of the cohort and enabling the identification of the most clinically relevant conditions.

Among the general characteristics of the population, the mean age was approximately 10 years higher than that reported in other studies [39]. Multimorbidity, frequent in geriatrics and associated with a significant impact on prognosis [35], was a clinically relevant finding: although multimorbidity in the strict sense (≥5 diseases) affected 13.3% of our sample, a burden of three or more chronic conditions was near-universal, as is typical of long-term care residents and implies a greater risk than in community-dwelling populations. For this reason, we analyzed multimorbidity defined as the presence of five or more diseases, an aspect already considered in formal geriatric assessments [34,36,40].

In the anthropometric parameters, BMI was above what would be expected for this type of population [41,42]. This parameter is an indicator with wide variability in the geriatric population and with limitations in reflecting real body composition. Regarding bioimpedance parameters, the observed body compositions may be below those reported in other populations [43,44,45]. These differences should be interpreted within the context of the type of population evaluated. Notably, phase angle presents one of the lowest mean values described in the literature. This finding may be explained by the high morbidity burden and the age of the cohort [44]. But what is noteworthy is that the differences are even more marked between those with chronic degenerative diseases compared to those without such conditions, reinforcing the relationship between disease burden and alterations in bioelectrical indicators.

### 3.1. Evaluation of Geriatric Syndromes

We analyzed four entities frequently observed in older adult patients. Three of these have been associated in the recent literature with the interpretation of physiological reserve [46,47,48]. In patients with chronic degenerative comorbidities, these entities play a relevant role in clinical prognosis [49,50], as they allow approximation of the individual’s degree of vulnerability. In both geriatrics and other specialties, the evaluation of these entities has been considered to reflect a possible estimation of physiological reserve; however, their prognostic capacity remains indirect [30,51].

Frailty has been consistently associated with increased morbidity and mortality [52,53], as well as with increased functional dependence and reduced physiological reserve. In our cohort, age alone was not associated as factor, which is consistent with evidence indicating that frailty does not depend strictly on chronological age, but rather on the accumulated burden of biological stress and functional deterioration [33]. In populations with a high comorbidity burden, expected vulnerability may be greater even among individuals of the same chronological age.

Frailty has been interpreted as a manifestation of reduced physiological reserve [27,28]. The FRAIL index proved appropriate for this population, as it integrates conceptual elements derived from both Fried’s physical phenotype and Rockwood’s cumulative deficit approach, allowing improved discrimination [34].

In this research, phase angle demonstrated the ability to identify this condition, with an area under the curve (AUC) of 0.718. This finding may be explained by the fact that phase angle directly reflects cellular membrane integrity and function, enabling detection of alterations related to systemic deterioration. Nevertheless, its performance is not absolute, suggesting the additional influence of other clinical variables in the expression of frailty.

Several studies have demonstrated that phase angle and other parameters derived from bioimpedance show good sensitivity for detecting sarcopenia [19,54,55,56]. This pathology has also been considered a marker of functional physiological reserve [15,31]. In our population, phase angle showed an association with sarcopenia, reinforcing the hypothesis about its usefulness as a complementary tool in the comprehensive evaluation of the elderly. This usefulness, however, is not uniformly supported. While many studies endorse the clinical value of phase angle, recent work has reported limited or inconsistent predictive performance; Piglowska et al., for instance, found a limited predictive value of bioelectrical phase angle for the development of sarcopenia in older Europeans [20]. Such discrepancies likely reflect differences in study populations, bioimpedance devices and measurement frequencies, and the absence of standardized, age and comorbidity adjusted cut-off values, reinforcing that phase angle should currently be regarded as a promising but not yet validated marker, to be confirmed across diverse populations and standardized protocols.

In addition, our approach holds that the interpretation of cellular health provides relevant information regarding the organism’s physiological reserve; in this sense, bioimpedance provides a parameter that reflects the amount of metabolically active body mass, or body cell mass.

When phase angle was combined with body cell mass, the area under the curve increased, yielding a higher apparent discriminative capacity (AUC 0.893), although this estimate is internally derived and requires confirmation. This interaction was not observed to the same extent in other entities, probably because the diagnosis of sarcopenia is based on specific physical parameters—muscle mass and function [41]—which are more directly related to cellular integrity.

Determining the patient’s degree of functionality and independence is fundamental in long-term care, as it is directly related to the identification of physiological [3,4,7] and functional reserve [57]. Various studies use the assessment of basic activities of daily living as an approach to estimate such reserve and guide therapeutic interventions [58,59]. In older adults in long-term care, functionality is one of the central objectives, with the aim of improving these scores to promote the greatest possible independence.

In this context, phase angle has shown promising results [60,61], demonstrating a significant and proportional correlation with the BI. It is also promising for patient follow-up, as phase angle has been documented to improve with physical rehabilitation [62] and regular physical activity [59,63], in parallel with increases in functional independence. Although it is not an absolute indicator and may be influenced by other clinical variables, its discriminative capacity across different levels of functionality supports its sensitivity.

Quality of life represents one of the central objectives in the care of patients in advanced stages of life. It is closely linked to the individual’s subjective perception of performance and well-being [64]. In the geriatric population, this perception can be affected by several aspects such as functional limitations, less social interaction, and barriers derived from comorbidity [65,66], which in turn introduces heterogeneity in its evaluation [67].

In this analysis, phase angle showed a relevant correlation with quality of life scores. This suggests that it could be employed as a screening tool to identify patients at risk of deterioration in this domain, allowing more in-depth evaluations and, eventually, preventive interventions.

### 3.2. Phase Angle as a Candidate Marker Associated with Physiological Reserve

Most investigations have sought to relate phase angle to the presence of a specific pathology [8,39,68]. Some authors went further, proposing that phase angle, together with metabolic equivalents, may serve as an indirect measure of the functional reserve of the organism, with lower values behaving as potential biomarkers of evolving frailty. That study, however, was conducted in fit community-dwelling older adults enrolled in a regular fitness program in which baseline reserve is largely preserved (only ~2% frail at entry) and used *functional reserve* as an interpretive label without defining or operationalizing the construct. Building on that observation, the present study reframes phase angle specifically as a quantifiable expression of physiological reserve at the cellular level and examines it in institutionalized long-term care residents, a population in which physiological reserve is markedly contracted and the margin to dependency is narrow. Functional and physiological reserve are overlapping, hierarchically related constructs—functional reserve being, in essence, the integrated behavioural expression of an underlying physiological substrate; our shift is therefore one of analytical level and population rather than a claim of conceptual disjunction [69].

Physiological reserve has been described in the literature [70,71] as the organism’s capacity to respond to stressors. The erosion of this capacity translates into vulnerability and manifests clinically in diagnoses such as frailty [27], and it explains why two patients of the same age may show markedly different responses to the same insult [3]. On this basis, and by integrating our findings with the existing evidence on bioimpedance, we advance a single hypothesis: that phase angle may serve as a numerical expression of physiological reserve, and therefore of vulnerability, in older adults.

The mechanistic rationale is direct. Phase angle is derived from the resistance and reactance measured when an alternating current is applied through the body, and its reactive component reflects the capacitive behaviour of cell membranes. A cell membrane behaves, in effect, as a biological capacitor: just as a capacitor’s ability to store and release charge declines over time, the cell does not abruptly lose function but undergoes a progressive loss of efficiency, and this decline is captured by the phase angle. Consistent with this, several studies—including ours—have documented that the parameter decreases with age [19,63] while simultaneously adapting to the patient’s comorbidity burden, which argues for the use of age adjusted reference ranges.

This membrane level rationale should not be overstated. Physiological reserve is a multidimensional construct that integrates cardiovascular, pulmonary, neuromuscular, endocrine, metabolic, immune and cognitive systems, and no single parameter can represent it in full. Phase angle captures one integrative axis of this construct—the integrity of cell membranes and the mass of metabolically active tissue—that plausibly underlies the performance of these systems, but it does not directly quantify organ-specific reserve. Alternative interpretations must therefore be kept in view: a low phase angle may also reflect shifts in the ECW/ICW ratio, malnutrition or systemic inflammation rather than reserve as such. Accordingly, we position phase angle as a complementary, integrative cellular marker, to be interpreted alongside—not in place of—established multidimensional geriatric assessment.

Our multivariable analysis, which given the sample size we regard as exploratory, suggested independent associations of both functionality and frailty with phase angle. Thus, although aging accounts for part of its variability, the parameter may also capture clinical dimensions that do not depend on age alone. This is coherent from a geriatric standpoint: chronological age is not the absolute determinant of clinical status, because aging unfolds in parallel with the accumulation of comorbidities, functional decline, and the progressive contraction of the organism’s capacity to respond, that is, vulnerability.

Taken together, phase angle reflects not only expected biological aging but also functional and frailty related components that behave independently of it. This integration supports the proposal that phase angle may serve as a candidate quantitative marker associated with physiological reserve in older adults, always interpreted in relation to age and clinical context.

Validating phase angle as such a marker will require prospective, longitudinal cohort studies that measure phase angle repeatedly and relate it to hard clinical outcomes—incident frailty and disability, hospitalization, and mortality—thereby establishing temporality, which a cross-sectional design cannot. External validation of the proposed cut-offs in independent long-term care and community-dwelling cohorts is equally necessary, ideally with adjustment for nutritional status, inflammation, hydration and renal function to isolate the independent contribution of phase angle. Our group is currently pursuing this line through studies using bioimpedance devices adapted for immobile patients and the development of a geriatric database of age and comorbidity adjusted reference values.

## 4. Limitations

This study has several limitations. First, its cross-sectional design precludes any causal or directional inference: a lower phase angle may reflect reduced physiological reserve, frailty and sarcopenia may themselves lower phase angle, or all three may be parallel consequences of underlying biological aging. These possibilities cannot be distinguished with simultaneously measured variables.

Second, with 45 participants and several predictors per model, the analyses are exposed to overfitting and limited statistical power; the number of events per variable fell below recommended thresholds in some models, so the reported estimates should be regarded as exploratory and potentially optimistic.

Third, multiple bioimpedance parameters were tested against several clinical outcomes without formal correction for multiple comparisons; some associations may therefore represent type I error and require independent confirmation.

Fourth, several determinants known to influence phase angle were not formally modelled as confounders. Some were partially captured but not entered in the models—nutritional status through anthropometric indicators (BMI, calf and arm circumference) and hydration through BIA-derived water compartments (ECW/ICW)—whereas systemic inflammation, renal function, and formal cognitive assessment were not evaluated. Although functional status, which partly reflects advanced cognitive decline, was included, no validated cognitive instrument (e.g., MMSE/MoCA) was applied. These factors should be incorporated and adjusted for future work.

Fifth, the study was conducted in a single long-term care institution with a very old, highly dependent population and among the lowest phase angle values reported in the literature; the proposed cut-offs may not generalize to community-dwelling older adults or other care settings.

Finally, all cut-off values were derived and evaluated within the same dataset, without external validation, and should be interpreted as exploratory rather than diagnostic thresholds. Consistent with this, our results are hypothesis-generating and intended to inform future longitudinal and mechanistic research.

## 5. Conclusions

Our findings, together with the existing literature, support a novel hypothesis regarding the assessment of geriatric patients: the continued exploration of phase angle as a candidate marker associated with physiological reserve in older adults. This parameter may, in the future, offer discriminatory capacity even in heterogeneous populations with a high burden of comorbidity, helping to identify differences in biological vulnerability.

An association was observed between phase angle and the presence of sarcopenia, functional status, and frailty, three conditions widely recognized as clinical manifestations of diminished physiological reserve. This relationship reinforces the need for further investigation and highlights its potential utility not only as a complementary diagnostic tool but also as an instrument for assessment and longitudinal monitoring.

We believe that, in the near future, the systematic incorporation of bioimpedance-based tools into geriatric assessment may contribute to improved detection, monitoring, and optimization of clinical care for older adults residing in long-term care facilities.

## Figures and Tables

**Table 1:** Baseline characteristics of the Study population and differences between participants with frailty and sarcopenia.

		FRAIL Index			EWGSOP2		
	All (*n* = 45)	Frailty (*n* = 23)	Without Frailty (*n* = 22)	*p*-Value	Sarcopenia (*n* = 29)	Without Sarcopenia (*n* = 16)	*p*-Value
Demographics							
Sex				0.054			0.222
Male (%)	12 (26.7)	9 (39.1)	3 (13.6)		6 (20.7)	6 (37.5)	
Female (%)	33 (73.3)	14 (60.9)	19 (86.4)		23 (79.3)	10 (62.5)	
Age $\bar{x}$ (SD)	87.51 (7.98)	88.7 (7.2)	86.3 (8.8)	0.314	89.5 (5.6)	83.9 (10.4)	0.061
Age ≥ 80 years (%)	38 (84.4)	20 (87.0)	18 (81.8)	0.474	28 (96.6)	10 (62.5)	0.005
Diaper use (%)	28 (62.2)	18 (78.3)	10 (45.5)	0.024	20 (69.0)	8 (50.0)	0.175
Paid caregiver (%)	36 (80.0)	22 (95.7)	14 (63.6)	0.009	26 (89.7)	10 (62.5)	0.039
Years in chronic care $\bar{x}$ (SD)	7.38 (5.94)	6.7 (4.2)	8.1 (7.4)	0.438	7.3 (4.8)	7.4 (7.8)	0.961
Multimorbidity (%)	6 (13.3)	6 (26.1)	0 (0)	0.022	5 (17.2)	1 (6.3)	0.292
Somatometry							
Height $\bar{x}$ (SD)	157.06 (9.94)	158.2 (11.0)	155.8 (8.7)	0.411	154.4 (8.8)	161.9 (10.4)	0.013
Weight $\bar{x}$ (SD)	68.32 (17.47)	69.3 (20.4)	67.3 (14.2)	0.71	62.1 (15.9)	79.7 (14.6)	0.001
BMI $\bar{x}$ (SD)	27.45 (5.55)	27.4 (6.7)	27.6 (4.1)	0.906	25.9 (5.6)	30.3 (4.4)	0.009
Abdominal circumference $\bar{x}$ (SD)	98.75 (20.33)	100.8 (24.2)	96.6 (15.5)	0.491	94.0 (19.0)	107.4 (20.4)	0.033
Hip circumference $\bar{x}$ (SD)	94.44 (7.51)	94.0 (8.6)	95.0 (6.4)	0.661	91.6 (7.5)	99.5 (4.2)	<0.001
Calf circumference $\bar{x}$ (SD)	31.26 (4.9)	30.2 (5.1)	32.2 (4.7)	0.249	29.4 (5.1)	34.3 (2.7)	0.001
Right thigh circumference $\bar{x}$ (SD)	44.50 (8.39)	42.4 (10.6)	46.7 (4.5)	0.086	42.3 (9.6)	48.6 (2.9)	0.014
Left thigh circumference $\bar{x}$ (SD)	45.46 (4.71)	44.4 (5.0)	46.6 (4.3)	0.119	44.0 (4.7)	48.1 (3.4)	0.004
Right arm circumference $\bar{x}$ (SD)	30.33 (7.12)	29.6 (9.2)	31.0 (4.2)	0.515	28.1 (7.2)	34.4 (5.0)	0.003
Left arm circumference $\bar{x}$ (SD)	31.07 (5.30)	31.0 (6.5)	31.2 (3.9)	0.88	29.4 (4.8)	34.1 (5.0)	0.003
Bioimpedance							
TBW $\bar{x}$ (SD)	28.37 (6.19)	28.9 (6.8)	27.8 (5.6)	0.556	25.8 (4.5)	33.1 (6.1)	<0.001
ICW $\bar{x}$ (SD)	16.67 (3.71)	16.9 (4.0)	16.5 (3.5)	0.733	15.1 (2.7)	19.6 (3.6)	<0.001
ECW $\bar{x}$ (SD)	11.70 (2.53)	12.0 (2.8)	11.3 (2.2)	0.346	10.7 (1.9)	13.5 (2.6)	0.001
Protein $\bar{x}$ (SD)	7.21 (1.61)	7.3 (1.7)	7.1 (1.5)	0.696	6.5 (1.2)	8.5 (1.6)	<0.001
Minerals $\bar{x}$ (SD)	3.03 (0.55)	3.1 (0.6)	2.9 (0.4)	0.165	2.9 (0.5)	3.3 (0.6)	0.019
BFM $\bar{x}$ (SD)	29.72 (11.72)	29.9 (14.2)	29.5 (8.7)	0.9	26.9 (11.3)	34.8 (10.9)	0.028
SLM $\bar{x}$ (SD)	36.04 (7.89)	36.7 (8.6)	35.4 (7.2)	0.582	32.7 (5.7)	42.1 (7.8)	<0.001
FFM $\bar{x}$ (SD)	38.61 (8.29)	39.4 (9.0)	37.8 (7.6)	0.545	35.2 (6.1)	44.9 (8.3)	<0.001
SMM $\bar{x}$ (SD)	19.75 (4.83)	20.0 (5.2)	19.5 (4.5)	0.717	17.7 (3.5)	23.5 (4.7)	<0.001
PBF $\bar{x}$ (SD)	42.10 (9.41)	41.0 (11.4)	43.2 (6.9)	0.436	41.5 (10.1)	43.2 (8.3)	0.583
BCM $\bar{x}$ (SD)	23.88 (5.32)	24.2 (5.7)	23.6 (5.0)	0.722	21.6 (3.8)	28.1 (5.2)	<0.001
BMC $\bar{x}$ (SD)	2.56 (0.46)	2.7 (0.5)	2.5 (0.4)	0.142	2.5 (0.4)	2.8 (0.5)	0.031
SMI $\bar{x}$ (SD)	5.34 (1.48)	5.2 (1.7)	5.5 (1.2)	0.413	4.7 (1.3)	6.5 (1.0)	<0.001
PhA° $\bar{x}$ (SD)	3.48 (0.87)	3.1 (0.7)	3.8 (0.9)	0.009	3.1 (0.7)	4.1 (0.9)	<0.001

Abbreviations: $\bar{x}$, mean; SD, standard deviation; BMI, body mass index; TBW, total body water; ICW, intracellular water; ECW, extracellular water; BFM, body fat mass; SLM, soft lean mass; FFM, fat-free mass; SMM, skeletal muscle mass; PBF, percent body fat; BCM, body cell mass; BMC, bone mineral content; SMI, skeletal muscle mass index; PhA, phase angle.

**Table 2:** Correlation analysis of population characteristics, functionality, and quality of life.

	Functionality		Quality of Life	
	Barthel Index	*p*-Value	WHOQOL OLD	*p*-Value
**Demographics**				
Sex $\bar{x}$ (SD)		0.674		0.427
Male	51.8 (30.6)		73.0 (12.4)	
Female	57.2 (38.7)		68.3 (15.0)	
Age *r*	−0.156	0.379	−0.251	0.158
Age ≥ 80 years $\bar{x}$ (SD)		0.629		0.022
No	59.2 (39.5)		75.8 (2.6)	
Yes	52.0 (31.4)		68.3 (15.3)	
Diaper use $\bar{x}$ (SD)		<0.001		0.001
No	80.4 (29.0)		78.4 (11.6)	
Yes	34.3 (18.2)		62.8 (12.7)	
Paid caregiver $\bar{x}$ (SD)		<0.001		<0.001
No	96.3 (6.9)		83.9 (5.8)	
Yes	40.0 (24.5)		64.8 (13.2)	
Years in chronic care *r*	0.007	0.967	−0.195	0.276
Multimorbidity $\bar{x}$ (SD)		0.714		0.416
No	53.6 (34.2)		68.6 (14.8)	
Yes	50 (14.7)		75 (9.8)	
**Somatometry**				
Height *r*	0.047	0.793	0.086	0.634
Weight *r*	−0.073	0.683	−0.131	0.467
BMI *r*	−0.114	0.519	−0.234	0.191
Abdominal circumference *r*	−0.255	0.146	−0.256	0.151
Hip circumference *r*	0.1	0.575	−0.071	0.693
Calf circumference *r*	0.176	0.380	0.090	0.662
Right thigh circumference *r*	0.478	0.004	0.266	0.134
Left thigh circumference *r*	0.449	0.008	0.216	0.228
Right arm circumference *r*	−0.083	0.639	−0.16	0.373
Left arm circumference *r*	−0.088	0.619	−0.176	0.326
**Bioimpedance**				
TBW *r*	0.171	0.334	0.154	0.391
ICW *r*	0.199	0.26	0.166	0.357
ECW *r*	0.125	0.48	0.134	0.458
Protein *r*	0.191	0.279	0.159	0.376
Minerals *r*	−0.013	0.943	0.077	0.671
BFM *r*	−0.212	0.228	−0.286	0.106
SLM *r*	0.176	0.32	0.156	0.386
FFM *r*	0.165	0.352	0.151	0.401
SMM *r*	0.195	0.269	0.164	0.363
PBF *r*	−0.156	0.377	−0.286	0.107
BCM *r*	0.196	0.265	0.164	0.363
BMC *r*	−0.03	0.865	0.059	0.745
SMI *r*	0.362	0.036	0.261	0.143
PhA° *r*	0.668	<0.001	0.551	0.001

Abbreviations: $\bar{x}$, mean; SD, standard deviation; *r*, correlation coefficient; BMI, body mass index; TBW, total body water; ICW, intracellular water; ECW, extracellular water; BFM, body fat mass; SLM, soft lean mass; FFM, fat-free mass; SMM, skeletal muscle mass; PBF, percent body fat; BCM, body cell mass; BMC, bone mineral content; SMI, skeletal muscle mass index; PhA, phase angle.

**Table 3:** Multiple linear regression model with PhA as the dependent variable.

Variables Included in the Model	B	β	*p*	95% CI for B
Age	−0.063	−0.620	<0.001	−0.077 to −0.048
Functionality	0.013	0.47	<0.001	0.008 to 0.017
Frailty	−0.137	−0.176	0.044	−0.270 to −0.004

Note: B (unstandardized coefficient); β (standardized coefficient); *p* (probability value); 95% CI for B (95% confidence interval for the unstandardized coefficient).

## Data Availability

The datasets used and/or analyzed during the current study are available from the corresponding author on reasonable request. The datasets generated and analyzed during the current study are not publicly available because they contain clinical information from vulnerable older adults residing in long-term care facilities and are subject to institutional and ethical restrictions regarding participant confidentiality. De-identified data may be made available by the corresponding author upon reasonable request and with permission from the Hospital Español de México Research and Ethics Committee.

## References

[1] Musich S., Wang S.S., Schaeffer J.A., Kraemer S., Wicker E., Yeh C.S. (2022). The association of increasing resilience with positive health outcomes among older adults. Geriatr. Nurs..

[2] Lima G.S., Figueira A.L.G., Carvalho E.C.d., Kusumota L., Caldeira S. (2023). Resilience in older people: A concept analysis. Healthcare.

[3] Hu F.W., Yueh F.R., Fang T.J., Chang C.M., Lin C.Y. (2024). Testing a Conceptual Model of Physiologic Reserve, Intrinsic Capacity, and Physical Resilience in Hospitalized Older Patients: A Structural Equation Modelling. Gerontology.

[4] Zhou Y., Ma L. (2022). Intrinsic Capacity in Older Adults: Recent Advances. Aging Dis..

[5] Nguyenhuy M., Chang J., Xu R., Virk S., Saxena A. (2022). The Fried Frailty Phenotype in Patients Undergoing Cardiac Surgery: A Systematic Review and Meta-Analysis. Heart Surg. Forum.

[6] Kim D.H., Rockwood K. (2024). Frailty in Older Adults. N. Engl. J. Med..

[7] Hajibandeh S., Hajibandeh S. (2023). Objective measurement of age-related physiological decline and vulnerability is still missing from the emergency laparotomy mortality predictive models. Anaesthesia.

[8] Sales W.B., Macedo S., Goncalves R., Andrade L.E.L., Ramalho C.S.T., de Souza G.F., Maciel A.C.C. (2024). Use of electrical bioimpedance in the assessment of sarcopenia in the older aldults: A scoping review. J. Bodyw. Mov. Ther..

[9] Pham M.D., Dao T.V., Vu A.T.X., Bui H.T.Q., Nguyen B.T., Nguyen A.T.T., Ta T.T.T., Cap D.M., Le T.D., Phan P.H. (2025). Association of Bioelectrical Impedance Analysis Parameters with Malnutrition in Patients Undergoing Maintenance Hemodialysis: A Cross-Sectional Study. Medicina.

[10] Quist J.R., Isidor S.D., Hvas C.L., Iversen P.R., Jodal L., Rud C.L., Brantlov S. (2025). Bioelectrical impedance analysis for assessing body composition. Ugeskr. Laeger.

[11] Lima J., Eckert I., Gonzalez M.C., Silva F.M. (2022). Prognostic value of phase angle and bioelectrical impedance vector in critically ill patients: A systematic review and meta-analysis of observational studies. Clin. Nutr..

[12] Zheng L., Li X., Xu Y., Yang Y., Wan X., Ma X., Yao G., Li G. (2025). Effects of Virtual Reality-Based Activities of Daily Living Rehabilitation Training in Older Adults With Cognitive Frailty and Activities of Daily Living Impairments: A Randomized Controlled Trial. J. Am. Med. Dir. Assoc..

[13] Sasegbon A., Weerasinghe P., Lal S. (2023). The relationships between sarcopenia, frailty, bioelectrical impedance analysis, and anthropometry in patients with type two intestinal failure. Clin. Nutr. ESPEN.

[14] Wu H., Ding P., Wu J., Yang P., Tian Y., Zhao Q. (2022). Phase angle derived from bioelectrical impedance analysis as a marker for predicting sarcopenia. Front. Nutr..

[15] Zhang J., Wang N., Li J., Wang Y., Xiao Y., Sha T. (2024). The Diagnostic Accuracy and Cutoff Value of Phase Angle for Screening Sarcopenia: A Systematic Review and Meta-Analysis. J. Am. Med. Dir. Assoc..

[16] Ito Y., Yoshimura Y., Nagano F., Matsumoto A., Wakabayashi H. (2024). Association of Phase Angle Dynamics with Sarcopenia and Activities of Daily Living in Osteoporotic Fracture Patients. Ann. Geriatr. Med. Res..

[17] Prete M., Ballarin G., Porciello G., Arianna A., Luongo A., Belli V., Scalfi L., Celentano E. (2024). Bioelectrical impedance analysis-derived phase angle (PhA) in lung cancer patients: A systematic review. BMC Cancer.

[18] Lukaski H.C., Garcia-Almeida J.M. (2023). Phase angle in applications of bioimpedance in health and disease. Rev. Endocr. Metab. Disord..

[19] Di Vincenzo O., Marra M., Di Gregorio A., Pasanisi F., Scalfi L. (2021). Bioelectrical impedance analysis (BIA) -derived phase angle in sarcopenia: A systematic review. Clin. Nutr..

[20] Piglowska M., Corsonello A., Kostka T., Roller-Wirnsberger R., Wirnsberger G., Arnlov J., Carlsson A.C., Tap L., Mattace-Raso F., Formiga F. (2024). Limited predictive value of bioelectrical phase angle for the development of sarcopenia in older Europeans. J. Nutr. Health Aging.

[21] Xian M., Yan Y., Lin J., Huang G., Xie K., Zeng D., Li L., Zhang Y. (2025). Phase angle: A novel application of bioelectrical impedance technology in osteoarthritis screening and diagnosis. Clin. Rheumatol..

[22] Popiolek-Kalisz J., Kalisz G., Zembala M. (2025). The Application of Bioelectrical Impedance Analysis Phase Angle in Cardiac Surgery. Nutrients.

[23] Ward L.C., Brantlov S. (2023). Bioimpedance basics and phase angle fundamentals. Rev. Endocr. Metab. Disord..

[24] Dellinger J.R., Johnson B.A., Benavides M.L., Moore M.L., Stratton M.T., Harty P.S., Siedler M.R., Tinsley G.M. (2021). Agreement of bioelectrical resistance, reactance, and phase angle values from supine and standing bioimpedance analyzers. Physiol. Meas..

[25] Mandala C., Veronese N., Dominguez L.J., Candore G., Accardi G., Smith L., Herrero M.T., Barbagallo M. (2023). Use of bioelectrical impedance analysis in centenarians: A systematic review. Aging Clin. Exp. Res..

[26] Kolodziej M., Ignasiak Z., Ignasiak T. (2021). Annual changes in appendicular skeletal muscle mass and quality in adults over 50 y of age, assessed using bioelectrical impedance analysis. Nutrition.

[27] Cosarderelioglu C., Walston J.D., Abadir P.M. (2025). From frailty to resilience: Exploring adaptive capacity and reserve in older adults-a narrative review. Front. Aging.

[28] Usui N., Nakata J., Uehata A., Kojima S., Ando S., Saitoh M., Inatsu A., Hisadome H., Nishiyama Y., Suzuki Y. (2025). Association of Physiological Reserve Obtained from Cardiopulmonary Exercise Testing and Frailty with All-Cause Mortality in Patients on Hemodialysis. Clin. J. Am. Soc. Nephrol..

[29] Wada O., Yamada M., Kamitani T., Mizuno K., Tadokoro K., Kurita N. (2023). Association between phase angle and functional disability among patients with lumbar spinal stenosis: The SPSS-OK study. J. Back. Musculoskelet. Rehabil..

[30] MacRae J.M., Harasemiw O., Lightfoot C.J., Thompson S., Wytsma-Fisher K., Koufaki P., Bohm C., Wilkinson T.J. (2023). Measurement properties of performance-based measures to assess physical function in chronic kidney disease: Recommendations from a COSMIN systematic review. Clin. Kidney J..

[31] Izquierdo M., Fiatarone Singh M. (2023). Promoting resilience in the face of ageing and disease: The central role of exercise and physical activity. Ageing Res. Rev..

[32] Boccardi V., Mancinetti F., Balducci C., Labbozzetta V., Ercolanetti E., Ruggiero C., Ercolani S., Mecocci P. (2026). Beyond the MNA: A Biological Vulnerability Phenotype Associated with Prolonged Hospitalization in Older Adults. Nutrients.

[33] Hanlon P., Wightman H., Politis M., Kirkpatrick S., Jones C., Andrew M.K., Vetrano D.L., Dent E., Hoogendijk E.O. (2024). The relationship between frailty and social vulnerability: A systematic review. Lancet Healthy Longev..

[34] Padilla Isassi A., Samra Saad A., Cervera Gaviria J., Chamlati Kemps M.P., Aguirre Dominguez J.A., Narvaez Valdivieso M.J. (2024). Frailty as a predictor of 3-year mortality in older adult patients in long-term care in Mexico. Rev. Esp. Geriatr. Gerontol..

[35] Tazzeo C., Zucchelli A., Vetrano D.L., Demurtas J., Smith L., Schoene D., Sanchez-Rodriguez D., Onder G., Balci C., Bonetti S. (2023). Risk factors for multimorbidity in adulthood: A systematic review. Ageing Res. Rev..

[36] Vetrano D.L., Palmer K., Marengoni A., Marzetti E., Lattanzio F., Roller-Wirnsberger R., Lopez Samaniego L., Rodriguez-Manas L., Bernabei R., Onder G. (2019). Frailty and Multimorbidity: A Systematic Review and Meta-analysis. J. Gerontol. A Biol. Sci. Med. Sci..

[37] Shiraishi R., Nakamura M., Araki S., Yokochi M. (2025). Evaluation of Trunk Muscle Mass in Older Adults Using Bioelectrical Impedance Analysis: A Scoping Review. JMA J..

[38] Konecna M., Poracova J., Sedlak V., Galova J., Babejova A., Zahatnanska M., Kimakova T., Nagy M., Bernatova R., Bernat M. (2023). Use of bioimpedance in prevention of sarcopenia in the elderly. Cent. Eur. J. Public Health.

[39] Jiang F.L., Tang S., Eom S.H., Lee J.Y., Chae J.H., Kim C.H. (2023). Distribution of Bioelectrical Impedance Vector Analysis and Phase Angle in Korean Elderly and Sarcopenia. Sensors.

[40] Forman D.E., Maurer M.S., Boyd C., Brindis R., Salive M.E., Horne F.M., Bell S.P., Fulmer T., Reuben D.B., Zieman S. (2018). Multimorbidity in Older Adults With Cardiovascular Disease. J. Am. Coll. Cardiol..

[41] Volkert D., Beck A.M., Cederholm T., Cruz-Jentoft A., Goisser S., Hooper L., Kiesswetter E., Maggio M., Raynaud-Simon A., Sieber C.C. (2019). ESPEN guideline on clinical nutrition and hydration in geriatrics. Clin. Nutr..

[42] Donini L.M., Busetto L., Bischoff S.C., Cederholm T., Ballesteros-Pomar M.D., Batsis J.A., Bauer J.M., Boirie Y., Cruz-Jentoft A.J., Dicker D. (2022). Definition and diagnostic criteria for sarcopenic obesity: ESPEN and EASO consensus statement. Obes. Facts.

[43] Rabelo I.F., Farrell S., Reid K.F., Dos Santos V.R., Antunes M., Batista V.C., Bauermann-Vieira A., Gobbo L.A. (2025). Bioelectrical impedance vectors analysis (BIVA) in older adults according to level of physical activity and muscle strength: A comparison of classic and specific approaches. Front. Aging.

[44] Campa F., Annunziata G., Barrea L., Sampieri A., Ceolin C., De Rui M., Sguaizer F., Petri C., Spataro F., Mascherini G. (2025). Bioelectrical impedance vector analysis in older adults: Reference standards from a cross-sectional study. Front. Nutr..

[45] Tomasiewicz A., Targowski T., Makuch S., Polanski J., Tanski W. (2025). The Relationship Between Bioelectrical Impedance Analysis Parameters and Laboratory Biomarkers in an Elderly Polish Cohort: A Cross-Sectional Study. Nutrients.

[46] Kimber J.S., Woodman R.J., Narayana S.K., John L., Ramachandran J., Schembri D., Chen J.W.C., Muller K.R., Wigg A.J. (2022). Association of physiological reserve measures with adverse outcomes following liver transplantation. JGH Open.

[47] Park J., Lee J., Lee H., Kim S., Kim C.O., Park C.G. (2022). Physical resilience as a moderator of the relationship between frailty and disability in older adults with osteoarthritis. J. Adv. Nurs..

[48] Walston J., Varadhan R., Xue Q.L., Buta B., Sieber F., Oni J., Imus P., Crews D.C., Artz A., Schrack J. (2023). A Study of Physical Resilience and Aging (SPRING): Conceptual framework, rationale, and study design. J. Am. Geriatr. Soc..

[49] Bhanji R.A., Watt K.D. (2021). Physiologic Reserve Assessment and Application in Clinical and Research Settings in Liver Transplantation. Liver Transpl..

[50] Vogelbaum M.A., Brown P.D., Messersmith H., Brastianos P.K., Burri S., Cahill D., Dunn I.F., Gaspar L.E., Gatson N.T.N., Gondi V. (2022). Treatment for Brain Metastases: ASCO-SNO-ASTRO Guideline. J. Clin. Oncol..

[51] Bogataj S., Mesaric K.K., Pajek M., Petrusic T., Pajek J. (2022). Physical exercise and cognitive training interventions to improve cognition in hemodialysis patients: A systematic review. Front. Public Health.

[52] Begue G., Roshanravan B. (2025). From Frailty to Fitness: Unraveling Mortality Risk in ESKD. Clin. J. Am. Soc. Nephrol..

[53] Liu L., Jin J., Zhang D., Cheng M., Feng L., Zhang Y., Hu C., Zhang S. (2026). Changes in frailty and all-cause mortality in patients on hemodialysis: A prospective cohort study. BMC Nephrol..

[54] Anusitviwat C., Vanitcharoenkul E., Chotiyarnwong P., Unnanuntana A. (2023). Dual-Frequency Bioelectrical Impedance Analysis is Accurate and Reliable to Determine Lean Muscle Mass in The Elderly. J. Clin. Densitom..

[55] Kilic M.K., Kizilarslanoglu M.C., Arik G., Bolayir B., Kara O., Dogan Varan H., Sumer F., Kuyumcu M.E., Halil M., Ulger Z. (2017). Association of Bioelectrical Impedance Analysis-Derived Phase Angle and Sarcopenia in Older Adults. Nutr. Clin. Pract..

[56] Norman K., Herpich C., Muller-Werdan U. (2023). Role of phase angle in older adults with focus on the geriatric syndromes sarcopenia and frailty. Rev. Endocr. Metab. Disord..

[57] Hu F.W., Lin C.H., Yueh F.R., Lo Y.T., Lin C.Y. (2022). Development and psychometric evaluation of the Physical Resilience Instrument for Older Adults (PRIFOR). BMC Geriatr..

[58] Brekke-Kumley B., Chebaro K., Cler K., Fox M., Lather M., Balusu C., Kinder P.R. (2025). Functional Outcomes After Reoperation for Recurrent Glioma: A Systematic Review and Meta-Analysis of Karnofsky Performance Status with Descriptive Health-Related Quality-of-Life Reporting. Cancers.

[59] Perez-Dominguez B., Casana-Granell J., Garcia-Maset R., Garcia-Testal A., Melendez-Oliva E., Segura-Orti E. (2021). Effects of exercise programs on physical function and activity levels in patients undergoing hemodialysis: A randomized controlled trial. Eur. J. Phys. Rehabil. Med..

[60] Costa Pereira J.P.D., Reboucas A.S., Prado C.M., Gonzalez M.C., Cabral P.C., Diniz A.D.S., Trussardi Fayh A.P., Silva F.M. (2024). Phase angle as a marker of muscle quality: A systematic review and meta-analysis. Clin. Nutr..

[61] Mascherini G., Zappelli E., Castizo Olier J., Leone B., Musumeci G., Totti V., Irurtia A., Roi G.S., Mosconi G., Sella G. (2020). Bioelectrical impedance vector analysis (BIVA) in renal transplant recipients during an unsupervised physical exercise program. J. Sports Med. Phys. Fit..

[62] Antunes M., Dos Santos L., Gobbo L.A., Silva A.M., Cunha P.M., Kassiano W., Cyrino L.T., Nabuco H.C.G., Rodrigues R.J., Sardinha L.B. (2025). Bioelectrical impedance vector analysis and phase angle in response to resistance training volume reduction in older women. Eur. J. Clin. Nutr..

[63] Campa F., Colognesi L.A., Moro T., Paoli A., Casolo A., Santos L., Correia R.R., Lemes I.R., Milanez V.F., Christofaro D.D. (2023). Effect of resistance training on bioelectrical phase angle in older adults: A systematic review with Meta-analysis of randomized controlled trials. Rev. Endocr. Metab. Disord..

[64] Matsuzawa R., Suzuki Y., Yamamoto S., Harada M., Watanabe T., Shimoda T., Yoshida A., Delgado C., Tamaki A., Matsunaga A. (2021). Determinants of Health-Related Quality of Life and Physical Performance-Based Components of Frailty in Patients Undergoing Hemodialysis. J. Ren. Nutr..

[65] Francis J., Baxter M., Giza D., Cheung K.L., Parks R. (2025). Patient-Reported Outcomes in Geriatric Oncology-Balancing Quality of Life and Therapeutic Effectiveness Using Primary Breast Cancer as a Model. Drugs Aging.

[66] Heess A., Meyer A.M., Becker I., Noetzel N., Verleysdonk J., Rarek M., Benzing T., Polidori M.C. (2022). The prognostic fingerprint of quality of life in older inpatients: Relationship to geriatric syndromes’ and resources’ profile. Z. Gerontol. Geriatr..

[67] Bajenaru L., Balog A., Dobre C., Draghici R., Prada G.I. (2022). Latent profile analysis for quality of life in older patients. BMC Geriatr..

[68] Hamada R., Tanabe N., Oshima Y., Yoshioka Y., Maetani T., Shiraishi Y., Sato A., Sato S., Ikeguchi R., Matsuda S. (2024). Phase angle measured by bioelectrical impedance analysis in patients with chronic obstructive pulmonary disease: Associations with physical inactivity and frailty. Respir. Med..

[69] Zanforlini B.M., Trevisan C., Bertocco A., Piovesan F., Dianin M., Mazzochin M., Alessi A., Zoccarato F., Manzato E., Sergi G. (2019). Phase angle and metabolic equivalents as predictors of frailty transitions in advanced age. Exp. Gerontol..

[70] Xing Y., Liu P., Yu W., Zhao Y., Wang Z., Wu Y., Gao X., Wang Y., Guo Y., Wang Y. (2025). Reliability and validity of the Clinical pHysical rEsilience assEssment Scale (CHEES) in community-dwelling older adults. Exp. Gerontol..

[71] Trică A., Golu F., Sava N.I., Licu M., Zanfirescu Ș.A., Adam R., David I. (2024). Resilience and successful aging: A systematic review and meta-analysis. Acta Psychol..

